# Supplementary figures and images for: The M-current works in tandem with the persistent sodium current to set the speed of locomotion
Source: PLoS Biol. 2020 Nov 13;18(11):e3000738. doi: 10.1371/journal.pbio.3000738 (PMC7688130; doi:10.1371/journal.pbio.3000738)

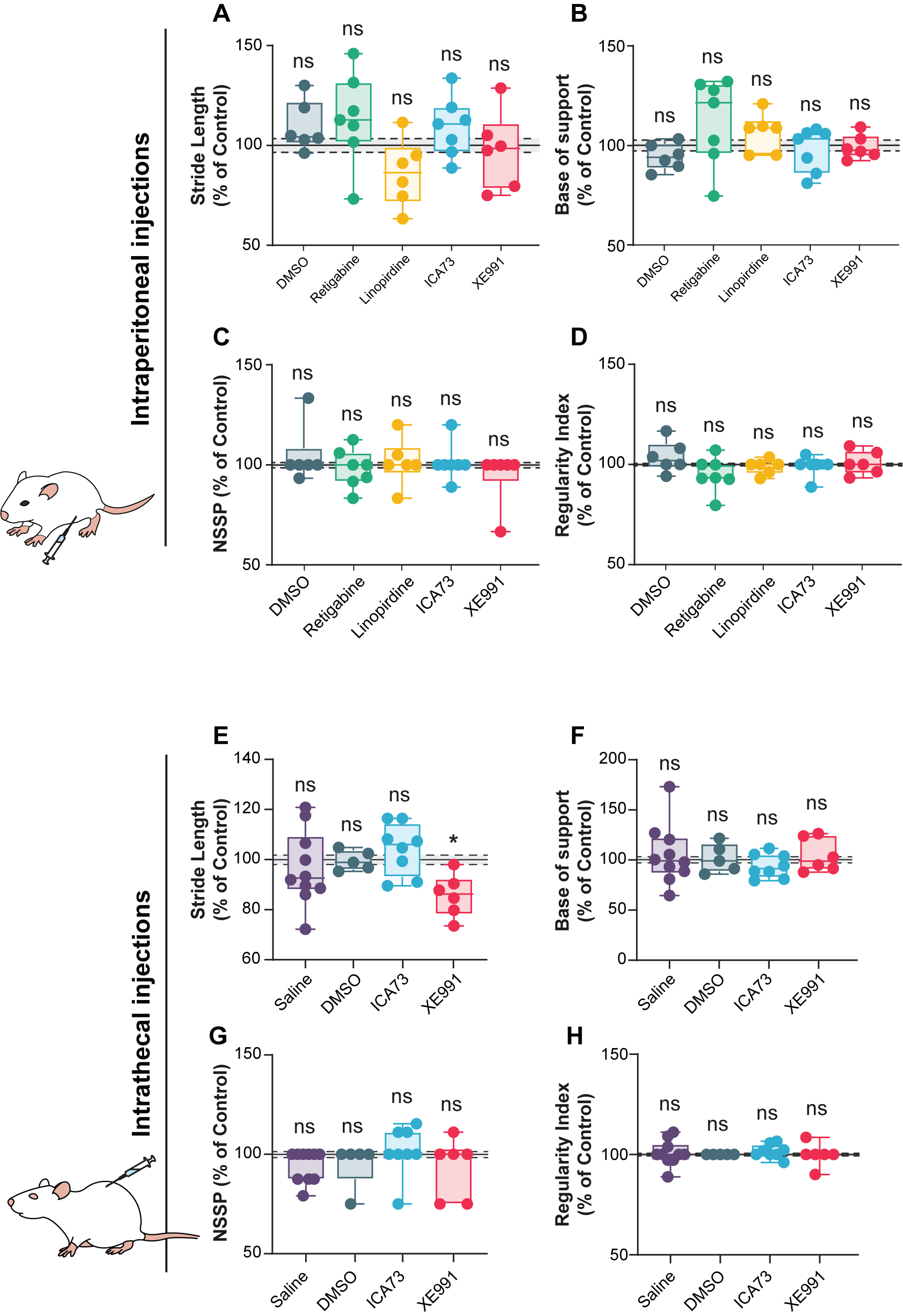

Supplement: S1 Fig — (A–H) Normalized changes of the stride length (A,E), the base of support (B,F), the number of normal step sequence patterns (C,G), and regularity index of paw placements (D,H) during CatWalk locomotion of juvenile rats (A–D) before and 30 min after acute i.p. administration of DMSO (gray, n = 6 rats), retigabine (5 mg/kg, green, n = 7 rats), linopirdine (3 mg/kg, yellow, n = 6 rats), ICA73 (5 mg/kg, blue, n = 7 rats), or XE991 (5 mg/kg, red, n = 6 rats) and of adult rats (E–H) before and 5–10 min after acute i.t. administration at the L1–L2 level of saline (purple, n = 10 rats), DMSO (gray, n = 5 rats), ICA73 (0.05 mg/kg, blue, n = 8 rats), or XE991 (0.025 mg/kg, red, n = 6 rats). Dashed lines with gray shading indicate the 95% confidence intervals of control values. ns, P > 0.05, comparing data collected before and after drug administration; Wilcoxon paired test. Underlying numerical values can be found in the S1 Data. ICA73, N-(2-chloro-5-pyrimidinyl)-3,4-difluorobenzamide; i.p., intraperitoneal; i.t., intrathecal; ns, not significant; XE991, 10,10-bis(4-pyridinylmethyl)-9(10H)-anthracenone dihydrochloride. (TIF) [file pbio.3000738.s001.tif]

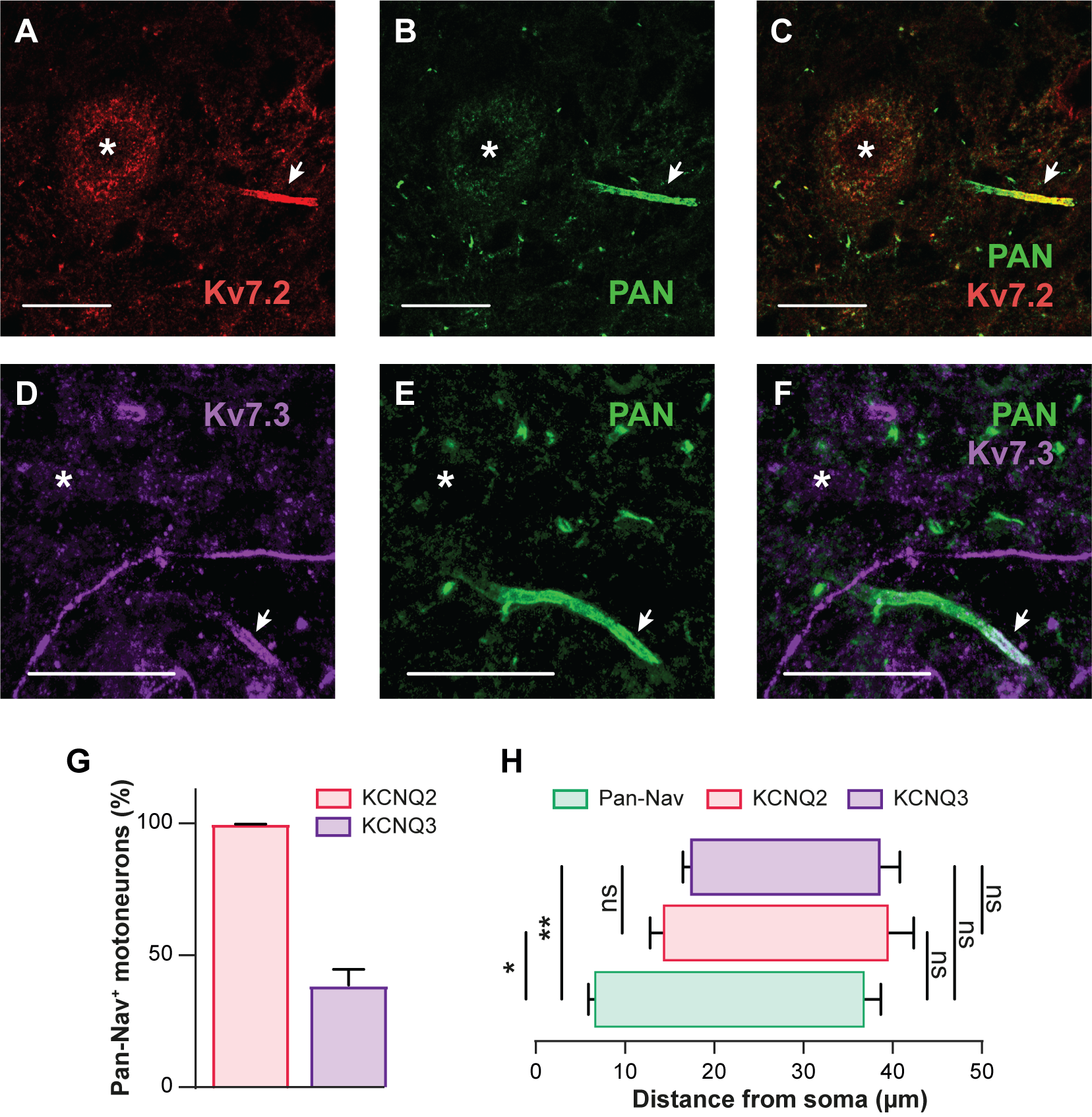

Supplement: S2 Fig — (A–F) Immunostaining of lumbar (L1–L2) motoneurons from juvenile rats (n = 3 rats) against Kv7.2 (A, n = 85 cells) or Kv7.3 (D, n = 104 cells) along the AIS labeled by the pan-Nav antibody (B,E). Kv7.2 and pan-Nav are merged in (C), and Kv7.3 and pan-Nav are merged in (F). Asterisks indicate the nucleus position and arrowheads the AIS. Scale bars = 20 μm. (G) Group means quantification of the proportion of pan-Nav positive motoneurons expressing Kv7.2 or Kv7.3 channels. (H) Group means quantification of the start and end positions of pan-Nav, Kv7.2, and Kv7.3 immunolabeling along the axonal process from the soma (n = 10 cells). *P < 0.05, **P < 0.01, comparing start or end positions between groups; Mann–Whitney test. Data are mean ± SEM. Underlying numerical values can be found in the S1 Data. AIS, axonal initial segment; Nav, voltage-gated sodium channel. (TIF) [file pbio.3000738.s002.tif]

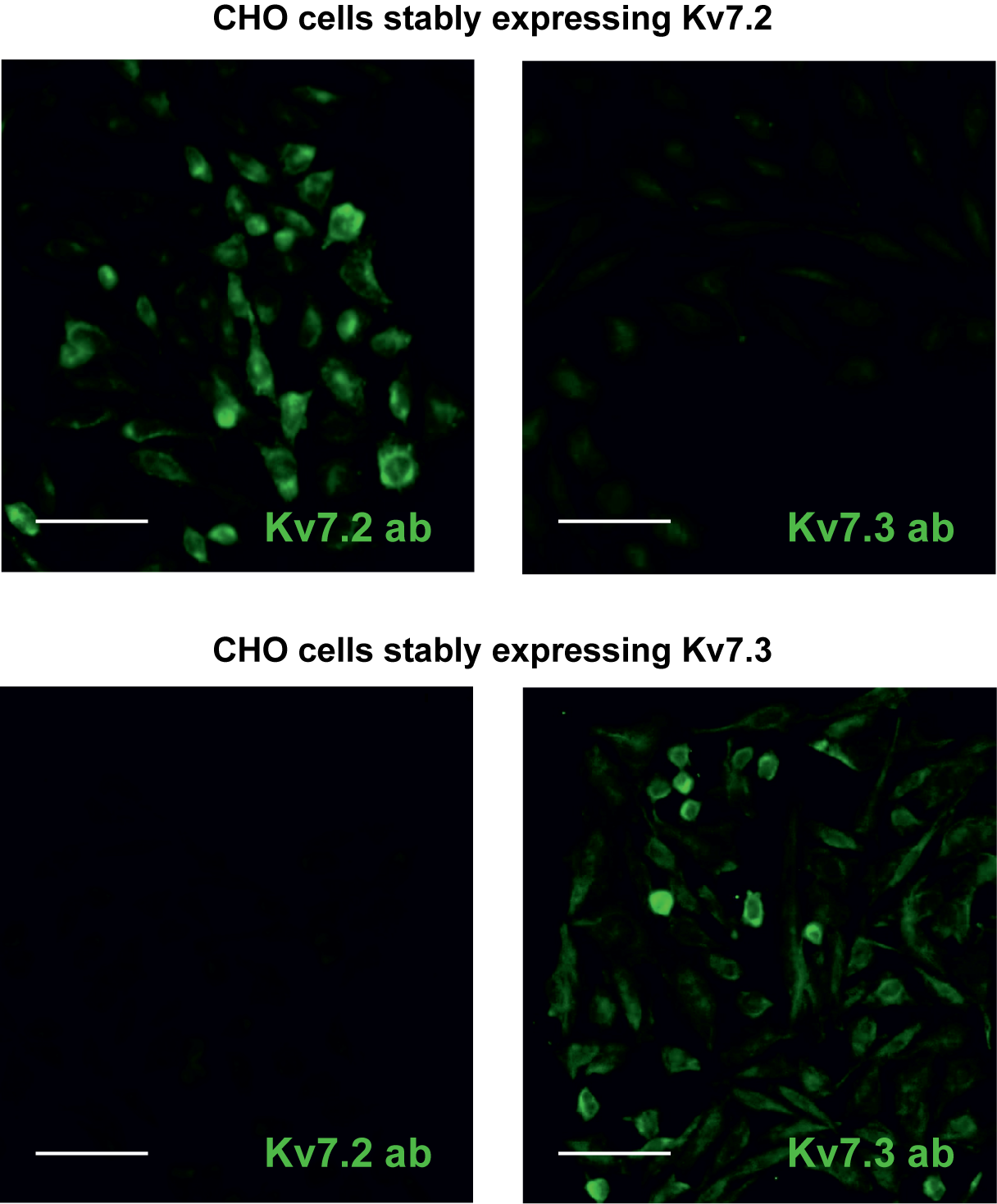

Supplement: S3 Fig — Immunostaining of Kv7.2-expressing (top) or Kv7.3-expressing (bottom) CHO cells in the presence of the Kv7.2 (left) or Kv7.3 (right) antibody. Scale bar: 50 μm. CHO, Chinese hamster ovary. (TIF) [file pbio.3000738.s003.tif]

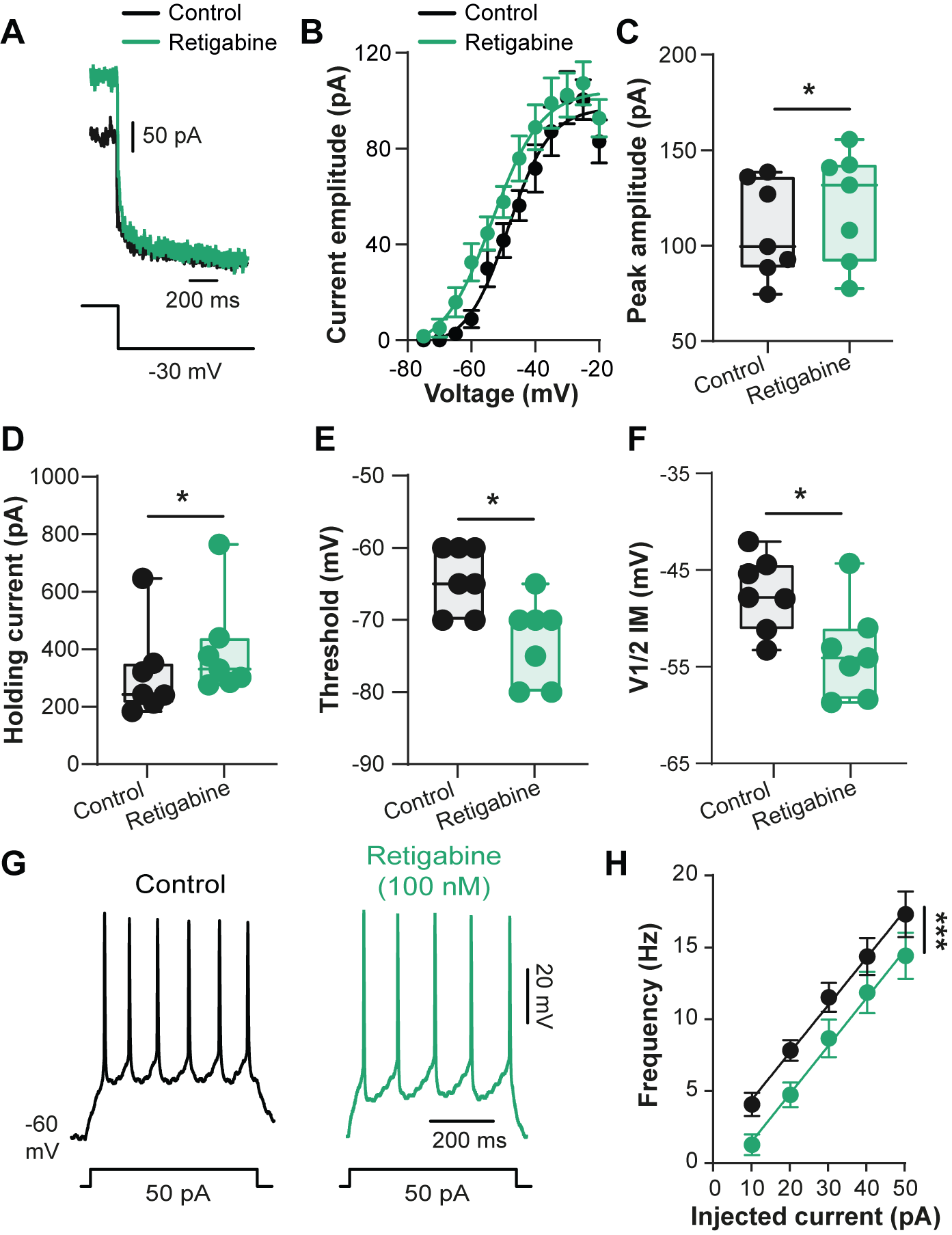

Supplement: S4 Fig — (A) Representative deactivation of IM. (B) Boltzmann-fitted I–V relationships of IM. (C–F) Boxplot quantification of the amplitude (C), holding current (D), threshold (E), and V1/2 (F) of IM recorded in interneurons (n = 7 cells) before (black) and after bath-applying retigabine (100 nM, green). *P < 0.05; Wilcoxon paired test. (G, H) Typical spiking activity of ventromedial interneurons (L1–L2) to a near-threshold depolarizing pulse (G) with the respective frequency–current relationship (H) before (black) and after bath-applying retigabine (100 nM, green). The continuous line is the best-fitting linear regression. ***P < 0.001, comparison of the fits. Underlying numerical values can be found in the S1 Data. ICA73, N-(2-chloro-5-pyrimidinyl)-3,4-difluorobenzamide; I–V, current–voltage. (TIF) [file pbio.3000738.s004.tif]

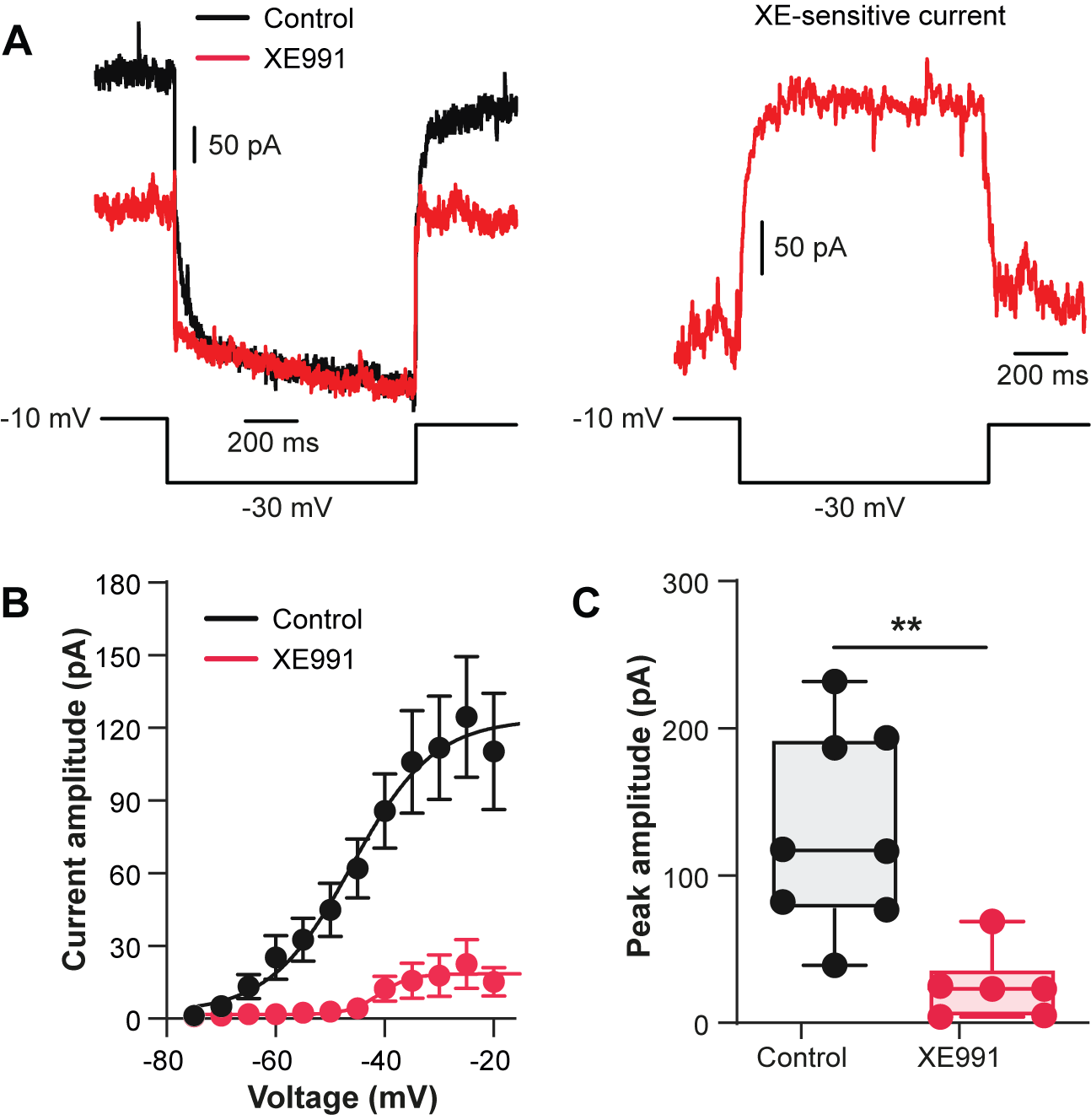

Supplement: S5 Fig — (A–C) Representative deactivation (A), Boltzmann-fitted I–V relationships (B), and amplitude (C) of IM recorded in Hb9+ interneurons from Hb9:eGFP transgenic neonatal mice under control conditions (n = 8 cells, black) or in the presence of XE991 (10 μM, n = 6 cells, red). *P < 0.05; Mann–Whitney test. Data are mean ± SEM. Underlying numerical values can be found in the S1 Data. eGFP, enhanced green fluorescent protein; I–V, current–voltage; XE991, 10,10-bis(4-pyridinylmethyl)-9(10H)-anthracenone dihydrochloride. (TIF) [file pbio.3000738.s005.tif]

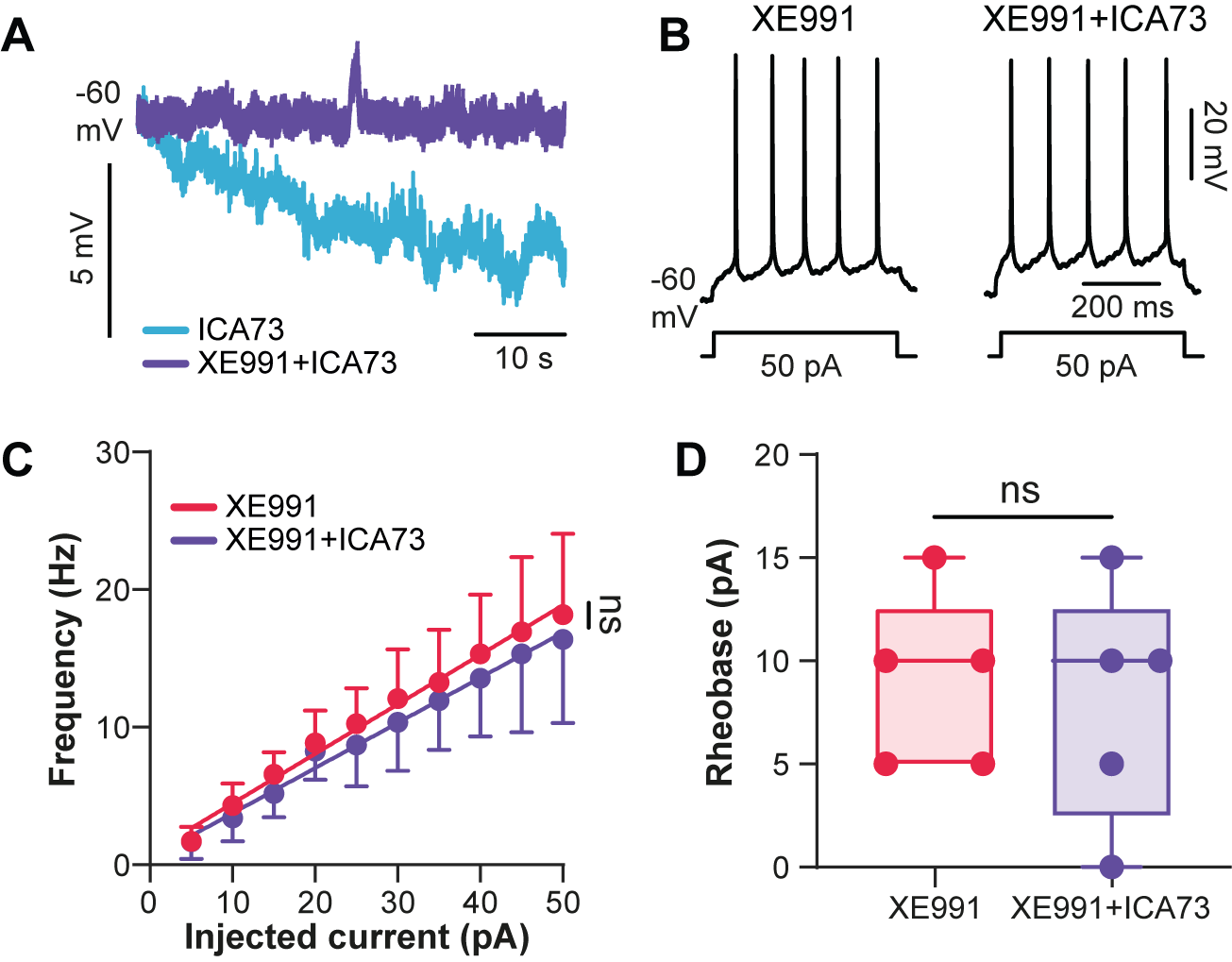

Supplement: S6 Fig — (A) Membrane potential changes in response to bath application of ICA73 (10 μM) before (light blue) and after (dark blue) pretreatment with XE991 (10 μM). (B,C) Representative spiking activity to a near-threshold depolarizing step (B) and frequency–current relationship (C) recorded under XE991 before (red) and after bath-applying ICA73 (n = 5 cells, dark blue). Continuous lines are the best-fitting linear regression. ns, P > 0.05 comparison of the fits. (D) Boxplot quantification of the rheobase. ns, P > 0.05; Wilcoxon paired test. Underlying numerical values can be found in S1 Data. ICA73, N-(2-chloro-5-pyrimidinyl)-3,4-difluorobenzamide; ns, not significant; XE991, 10,10-bis(4-pyridinylmethyl)-9(10H)-anthracenone dihydrochloride. (TIF) [file pbio.3000738.s006.tif]

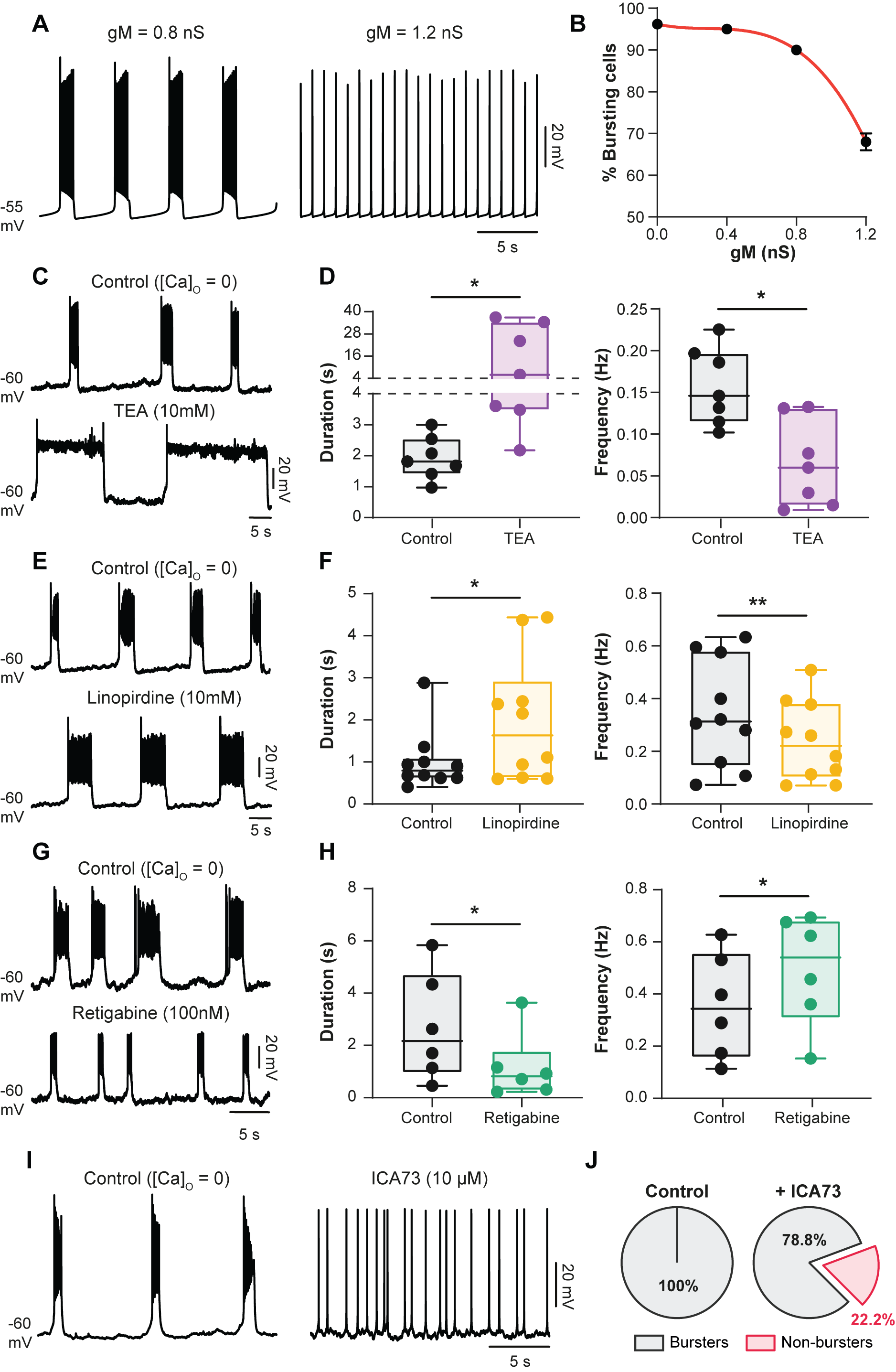

Supplement: S7 Fig — (A) Firing behavior from a bursting pacemaker neuron model in response to 2 different values of gM. (B) Dependence of the percentage of bursting cells on gM in the heterogeneous population model of 50 neurons. In A and B. V1/2 INaP = −54 mV. (C,E,G,I) [Ca2+]o-free-saline–induced bursting activity recorded intracellularly in L1–L2 ventromedial interneurons before and after TEA (10 mM, n = 7 cells) (C), linopirdine (10 μM, n = 10 cells) (E), retigabine (100 nM, n = 6 cells) (G), or ICA73 (10 μM, n = 9 cells) (I). (D,F,H) Boxplot quantification of the duration and frequency of bursts. *P < 0.05, **P < 0.01, comparing data before and after the abovementioned drugs; Wilcoxon paired test. (J) Proportion of burster and nonburster interneurons before and after bath-applying ICA73 (n = 9 cells). Underlying numerical values can be found in the S1 Data. ICA73, N-(2-chloro-5-pyrimidinyl)-3,4-difluorobenzamide; TEA, tetraethylammonium. (TIF) [file pbio.3000738.s007.tif]

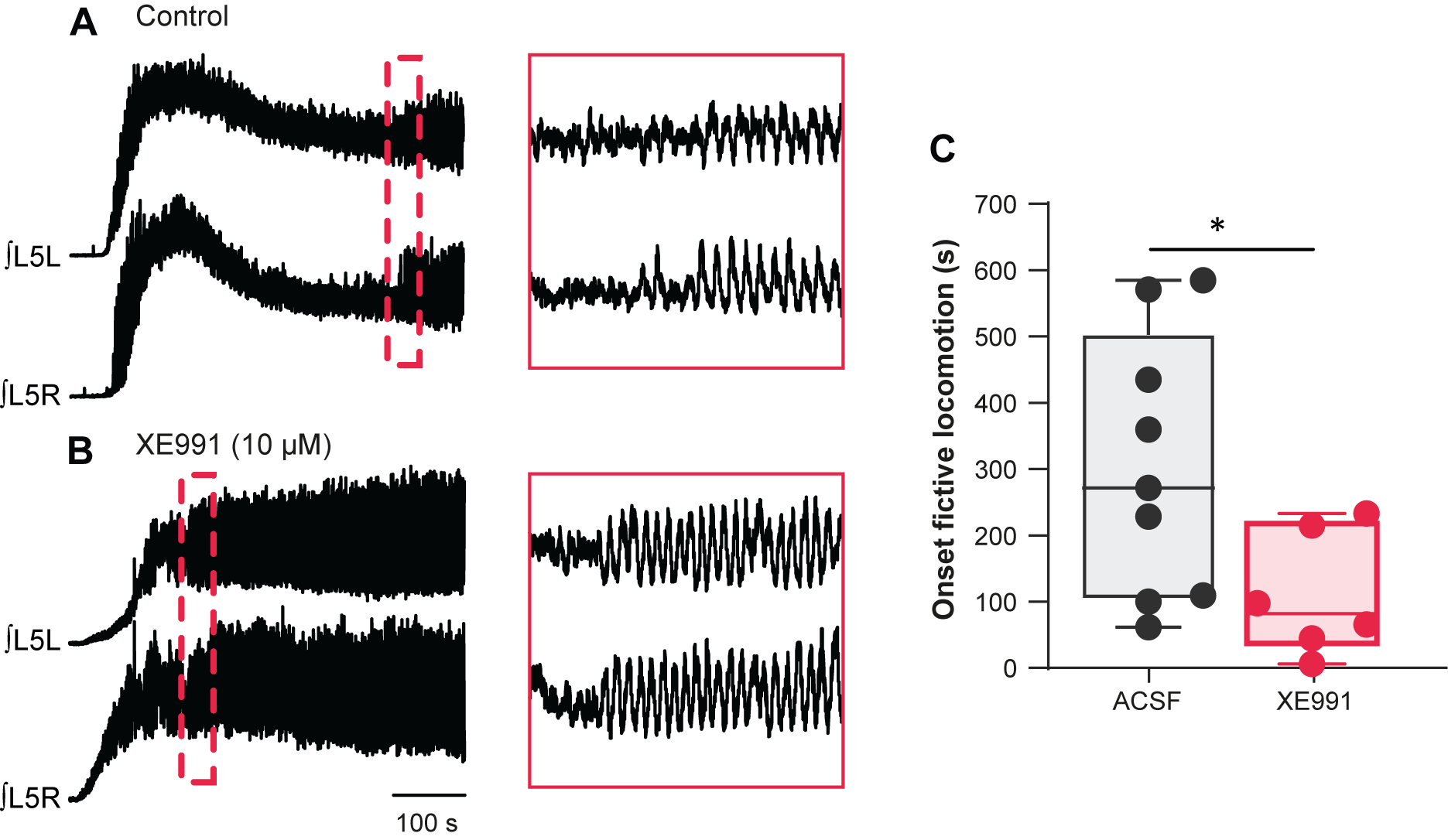

Supplement: S8 Fig — (A, B) Ventral-root recordings of NMA/5-HT–induced rhythmic activity generated without (n = 9 spinal cords) (A) and with (n = 6 spinal cords) a 45-min preincubation of XE991 (10 μM) (B). Broken-line boxes indicate the parts of the recordings that are enlarged in insets to visualize the onset of locomotor-like activity (rhythmic alternating activity). (C) Boxplot quantification of the delay between the start of the bath application of NMA/5-HT and the onset of the fictive locomotion. *P < 0.05; Mann–Whitney test. Underlying numerical values can be found in the S1 Data. NMA, N-methyl-DL aspartate; XE991, 10,10-bis(4-pyridinylmethyl)-9(10H)-anthracenone dihydrochloride; 5-HT, 5-hydroxytryptamine. (TIF) [file pbio.3000738.s008.tif]

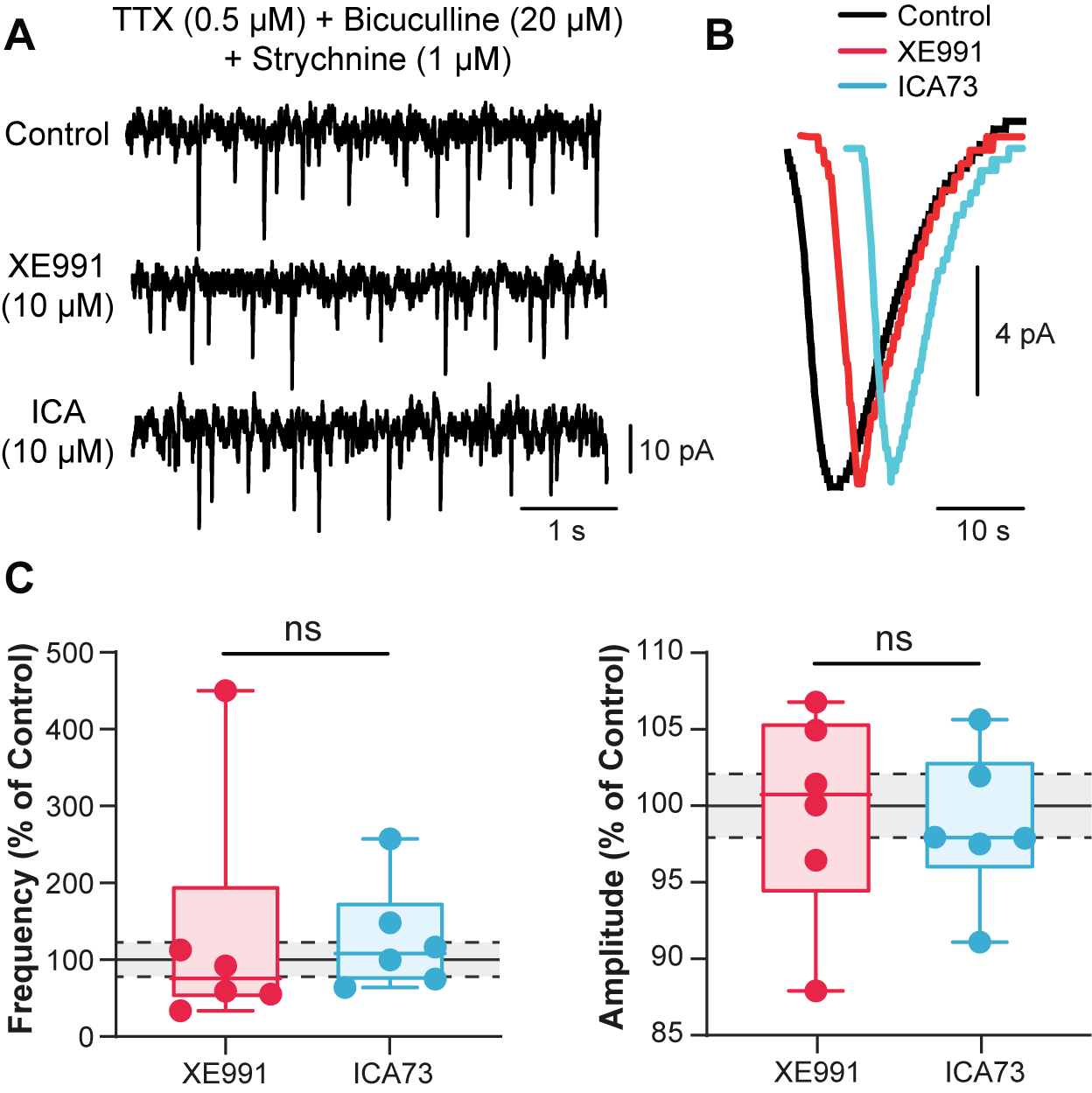

Supplement: S9 Fig — (A) Representative current traces of continuously recorded mEPSCs in a ventromedial interneuron voltage clamped at −60 mV. mEPSCs recorded before and 30 min after adding XE991 (10 μM, n = 6 cells) or ICA73 (10 μM, n = 6 cells) were pharmacologically isolated in the presence of TTX (0.5 μM), strychnine (1 μM), and bicuculline (20 μM). (B) Averaged mEPSCs recorded before (black) and 30 min after adding XE991 (red) or ICA73 (blue). (C) Boxplot quantification of the mean frequency and amplitude of mEPSCs before and 30 min after XE991 or ICA73 was bath-applied. Dotted lines indicate the 95% confidence intervals of control values. ns, P > 0.05, comparing data collected before and after bath-applying the abovementioned drugs; Wilcoxon paired test. Underlying numerical values can be found in the S1 Data. ICA73, N-(2-chloro-5-pyrimidinyl)-3,4-difluorobenzamide; mEPSC, miniature excitatory postsynaptic current; ns, not significant; TTX, tetrodotoxin; XE991, 10,10-bis(4-pyridinylmethyl)-9(10H)-anthracenone dihydrochloride. (TIF) [file pbio.3000738.s009.tif]

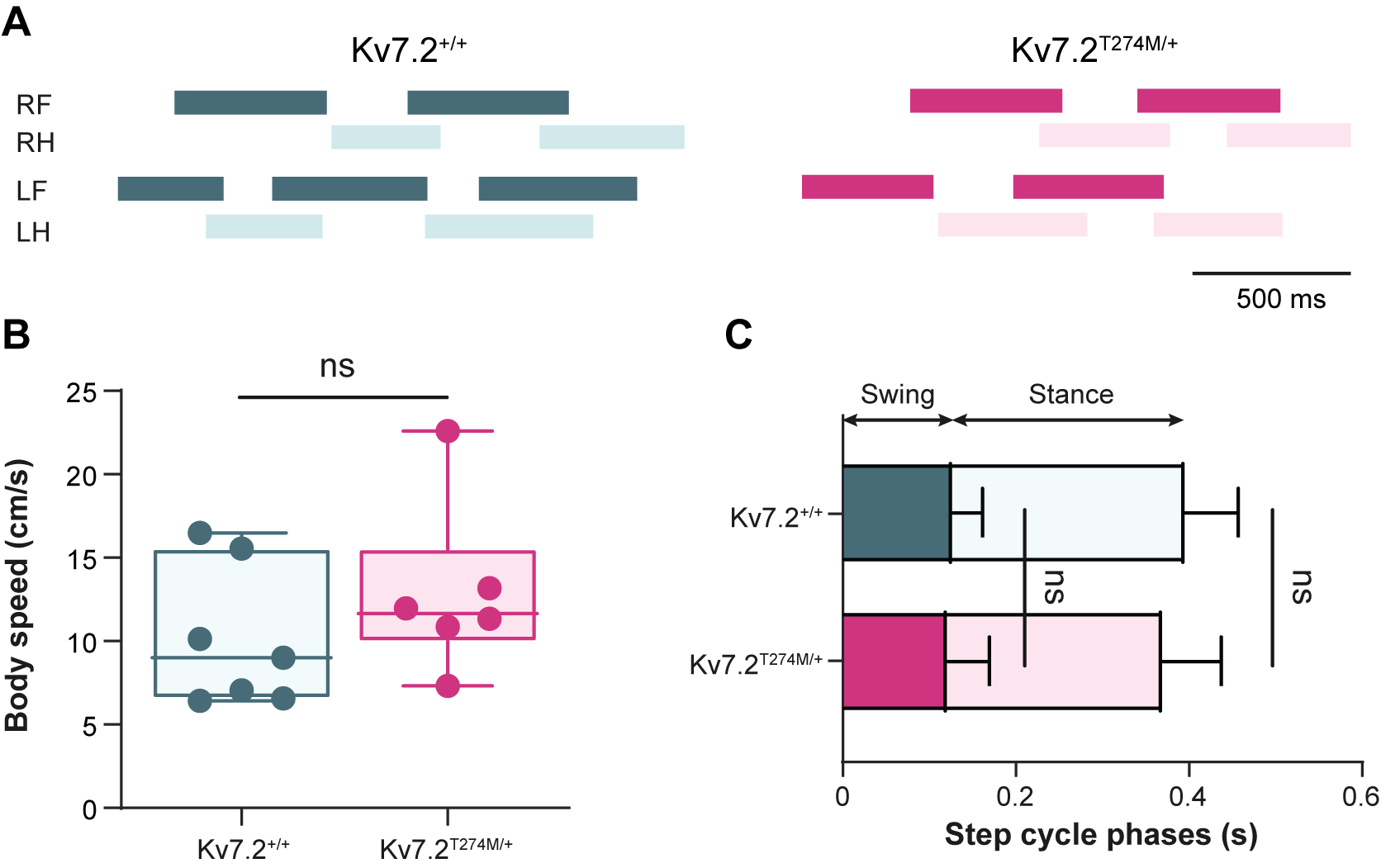

Supplement: S10 Fig — (A) Representative footfall diagrams during CatWalk locomotion of juvenile wild-type (gray, n = 7 mice) or Kv7.2Thr274Met/+ mutant mice (pink, n = 6 mice). The stance phase is indicated by horizontal bars and the swing phase by open spaces. (B) Boxplot quantification of the body speed. ns, P > 0.05, comparing wild-type versus Kv7.2Thr274Met/+ mutant mice; Mann–Whitney test. (C) Quantification of swing and stance phases. ns, P > 0.05, comparing wild-type versus Kv7.2Thr274Met/+ mutant mice Mann–Whitney test. Data in C are mean ± SEM. Underlying numerical values can be found in the S1 Data. LF, left forelimb; LH, left hindlimb; ns, not significant; RF, right forelimb; RH, right hindlimb. (TIF) [file pbio.3000738.s010.tif]
