# Supplementary material for: The M-current works in tandem with the persistent sodium current to set the speed of locomotion
Source: PLoS Biol. 2020 Nov 13;18(11):e3000738. doi: 10.1371/journal.pbio.3000738 (PMC7688130; doi:10.1371/journal.pbio.3000738)
Supplement: Attachment — Submitted filename: Responses to reviewers.docx [file pbio.3000738.s012.docx]

Manuscript Number: PBIOLOGY-D-20-01097R1

**Title: It takes two to tango: M-current swings with the persistent** **sodium current to set the speed of locomotion.**

Authors: Jeremy Verneuil, Cécile Brocard, Virginie Trouplin, Laurent Villard, Julie Peyronnet-Roux and Frédéric Brocard.

We would like to thank the reviewers for their helpful suggestions and the editors for their interest regarding the manuscript. We have significantly improved the manuscript and performed additional experiments and analyses to fully address the reviewers concerns. Please find below a point-by-point response.

**Reply:**

**Reviewer #1, Tuan Bui: The spinal cord has the remarkable ability to generate organized, sustained rhythmic activity underlying locomotor activity. However, the operation of spinal locomotor networks is not fully understood. This study uses a combination of behavioural testing, immunohistochemistry, pharmacology, computational modelling, and electrophysiology in a number of spinal neurons and spinal cord preparations to show that the subthreshold non-inactivating potassium M-current acts as a break to counterbalance the persistent sodium current that has been previously shown to be one of the principal rhythmogenic drive of spinal locomotor networks. Using both enhancers and blockers of Kv7 channels or their sub-units as well as transgenic mice with mutations to the gene encoding Kv7.2, the study characterizes this current in spinal neurons and shows that this current terminates bursts in spinal neurons. Their computational modelling allows them to support the conclusions about the role of Kv7 channels inferred from their experimental work**

**I have no major issues with the study. It provides invaluable insights that will further our understanding of the operation of spinal locomotor networks by opening lines of investigations into how this current is modulated by spinal and supraspinal pathways to regulate locomotor activity.**

**Minor issues:**

**1. Page 9, Fig 4 data, does the model replicate the translation of the I-F curve by enhancement of the M-current without a change in slope? Or if it did not because Kv7 inhibitors are less efficient at hyperpolarized membrane potential, could a model of M-current where the M-current is blocked at more depolarized membrane potential to replicate the potential-dependance of the inhibitors show the same changes in the I-F curves?**

The increase of the M-conductance (*g*_M_) in the model captured the modulation of neuronal excitability observed experimentally with I_m_-enhancers; i.e. an increased rheobase and a decreased firing rate (see new Fig 4F and 4G). Thus, the enhancement of *g*_M_ in the model replicated the shift of the f-I curve to the right without affecting the slope. By contrast, the decrease of *g*_M_ in the model produced opposite effects and predicted that the firing rate and the rheobase went up and down, respectively (see new Fig 4F and 4G). Thus, the decrease of *g*_M_ in the model shifted the f-I curve to the left. These data from the model are now illustrated in the Figure 4G. Furthermore, similar results were obtained in Kv7.2^Thr274Met/+^ mutant mice and are now also illustrated (see new Fig. 4C and 4D).

**2. Page 10, line 3, could you estimate the percentage of sampled ventromedial interneurons where blocking the M-current enhanced a iNaP? Fig. 4 G-H suggests that this might only be found in 18.2% of the population.**

Unfortunately, our recording conditions do not allow us to answer the question. In this set of experiments, we did not characterize the persistent sodium current (I_Nap_) within the same cell before and after the application of XE991. We sampled I_Nap_ in two different groups of neurons, one in the presence of XE991 and the other without XE991. It is likely that most of interneurons express a stronger I_Nap_ in the presence of the Kv7-blocker. However, this increase in I_Nap_ current does not mean that it will systematically lead to the genesis of bursts.

**3. Page 11, first paragraph, was a prolongation of the interburst by M-current blockade found in either the in-vitro locomotor activity with M-current blockade or the mutant KCNQ line?**

Yes, the interburst duration recorded during fictive locomotion was also increased when I_M_ was pharmacologically or genetically reduced. An opposite effect was observed when I_M_ was enhanced. This parameter is now quantified, added in results and illustrated in the figure 8.

**4. Page 11, line 24, missing "of" between "blockade" and "Im"**

Correction was done in the text.

**5. Page 12, line 5 "converted rhythmic bursting to spiking into few cells". Do you mean "converted rhythmic bursting into tonic spiking in a few cells"?**

Yes. Changes have been done appropriately in the text to avoid any confusions.

**6. Page 14, line 17, remove Pan.**

Correction was done in the text.

**7. Page 28, line 10, change "depended" to "dependent.**

Correction was done in the text.

**8. Page 29. The time constants of the peak of the m-variable for both the K+ delayed rectifier and of the M current should be in seconds not in mV.**

Correction was done in the text.

**Reviewer #2, Andrea Nistri, SISSA, Trieste: The MS by Verneuil et al. reports an interesting study of the role of the M current (IM) in the operation of the spinal networks responsible for locomotion. These data are novel and of general interest as they indicate important molecular and biophysical components of a circuitry that relays on the interplay amongst a number of ionic conductances expressed by key neurons to generate walking. The present study is multidisciplinary and well organized and requires a relatively small degree of attention to improve it.**

**Although the MS is overall clearly written, the authors are advised to improve the English style and to proofread the text in order to eliminate editing errors such as having names of quoted references alongside the ref. number.**

**It is suggested that the editor should check if there is a suitable repository for the original experimental data.**

1) **The authors state that "a decrease of IM converts a spiking pattern into a bursting mode in a subset of ventromedial spinal interneurons" (p. 15). How would this occur physiologically? What cells would release ACh? Information on this subject would be useful.**

The point about the cholinergic neuromodulation of the locomotor network related to the muscarinic inhibition of *I_M_* has been further discussed as following (see page 19 line 17 to page 20 line 8 in tracked manuscript) “*I_M_ is a well-established target for a range of modulators (Marrion, 1997; Selyanko et al., 2000; Delmas and Brown, 2005; Greene and Hoshi, 2017), and thus may offer a powerful mean to regulate the rhythmicity of the spinal locomotor network. Regarding the initial characterization of I_M_ through its suppression by muscarinic receptor activation (Brown and Adams, 1980; Adams and Brown, 1982) one obvious candidate is acetylcholine. Because the inhibition of I_M_ slowed down the locomotor rhythm, the cholinergic inhibition of I_M_ at the CPG level might play a key role in the dynamic reconfiguration of the locomotor network by changing the relative number of bursting pacemaker cells. (Purvis et al., 2007). In line with this notion: 1) the mammalian spinal cord contains several types of cholinergic neurons (Barber et al., 1984) 2) facilitation of the endogenous cholinergic system is capable of producing fictive locomotor activity in a slow speed range with a significant contribution of muscarinic receptors (Kiehn et al., 1996; Anglister et al., 2008; Etlin et al., 2014; Jordan et al., 2014). 3) cholinergic cells mainly located in laminae VII and near the central canal are recruited during locomotion (Huang et al., 2000; Miles et al., 2007; Zagoraiou et al., 2009; Tillakaratne et al., 2014) and 4) a significant proportion of CPG neurons are responsive to acetylcholine in the form of intrinsic membrane potential oscillations (Kiehn et al., 1996; Carlin et al., 2006). Altogether, the neuromodulation of I_M_ through muscarinic receptors may account, at least in part, for the cholinergic locomotor rhythm control.*”

**2) The authors report that injection of retigabine increased the speed of rat walking behaviour. However, retigabine was used as a second line antiepileptic drug (now discontinued) and there are no reports of a similar effect on man.**

Indeed, we did not find any case report on this topic possibly because this has never been investigated in humans, likely due to the fact that it was not widely prescribed and that a fine analysis is required to highlight effects on locomotor movements.

**3) Since the patch pipette contained K-gluconate, it is important to indicate if the membrane potential values were corrected for junction potential.**

This point has been clarified in the data analysis section of material and methods.

**4) The Methods section should contain more details about the antibody specificity with suitable references (western blotting, KO animals etc).**

The antibody against K_V_7.3 (residues 668–686; 1:400, Alomone) has been previously validated in conditional knock-out (Kcnq3 cKO) mice in Soh et al., 2014 J. Neurosci. **34**, 5311. After checking in the bibliography and data sheet, the antibody against K_V_7.2 used in the present study (residues 1–70; 1:1000, Thermo Scientific) seems not to be formally validated.

To establish the specificity of the antibodies, we performed additional experiment using chinese hamster ovary (CHO) cells stably expressing Kv7.2 or Kv7.3 channels. The CHO cells stably expressing Kv7.2 were strongly immunostained with the K_V_7.2 antibody and no labeling above background level was observed with the Kv7.3 antibody (**S2 Fig**). Likewise, the CHO cells stably expressing Kv7.3 were immunostained with the K_V_7.3 antibody and no labeling above background level was observed with the Kv7.2 antibody (**S2 Fig**). In sum, these data provide solid evidences of the specificity of the antibodies against Kv7.2 and Kv7.3 channels with no cross-reactivity. These new data have been mentioned in results (see page 6 last line and page 7 first lines in tracked manuscript) and in methods (see page 29, lines 10-18in tracked manuscript) and also illustrated in a supplementary figure (see new S2 Fig). Protocols related to the use of CHO cells have been added in methods (see Page 27 lines 17-25; page 28, lines 22 to page 29 line 8 in tracked manuscript).

**5) The argument that TEA does not affect the M current should be thoroughly revised. There are numerous reports that even at 1 mM concentration TEA can inhibit this current. See for example: Freschi JE. J Neurophysiol. 1983;50:1460-78; Constanti A, Sim JA. J Physiol. 1987;387:173-94; Halliwell JV, Horne AL. J Physiol. 1995;487:421-40.**

There is likely a misunderstanding. We did not stipulate that TEA does not affect the M current. As we wrote in the original manuscript (see page 12, lines 19-20 in tracked manuscript) “*our result led us to consider the involvement of a non-inactivating* ***TEA-sensitive*** *K^+^-current such as I_M_*.”

**6) Importantly, previous studies have shown that the subunit composition of the M current determines the sensitivity to TEA. See Smith JS et al. J Neurosci. 2001;21:1096-103, and Shah M et al. J Physiol. 2002,544:29-37. These reports should be discussed in the light of the MS results.**

See answer to the major question 1 from the reviewer 4

**7) In general, the actual amplitude of the M current is not very large. It would be interesting if the authors could discuss a bit more the relative contribution of this conductance versus other K currents in determining bursting patterns.**

We agree that *I_M_* is a minor part of the burst firing-activated outward current contributing to the burst termination. Consistent with this, the substantial increase in burst duration induced by TEA assumes the contribution of others voltage-gated K^+^ currents in addition to *I_M_* in the burst termination process (see S7C Fig). This observation is in line with a previous report identifying at least three K^+^ conductances (*I*_A_; *I*_Kdr_; *I*_KCa_) controlling the bursting activity in cultured spinal neurons (Legendre et al., 1985). Note that in our recording conditions performed in [Ca^2+^]_o_-free saline, no obvious role could be attributed to *I*_KCa_. On the other hand, because bursting activities rely on I_NaP_, outward currents coupled to Na^+^ accumulation such as Na^+^/K^+^ ATPase electrogenic pump current or Na^+^-dependent K^+^ current might be candidates for assisting burst termination. These two conductances have been shown to contribute to terminating bursts in the context of motor rhythm generation (Krey et al., 2010). These aspects are now added in the discussion (see Page 18 line 16 to page 19 line 5 in tracked manuscript)

**Reviewer #3: General Comments**

**This is an interesting paper by an excellent group on the interaction between Kv7 channels and persistent sodium currents. The group has published several findings on the role of persistent inward currents and their role in locomotor activity. This is the first paper to examine the interesting interactions that occur between currents that appear to operate together in regards to burst dynamics. They first show the effects of M-current manipulation in vivo and then through a series of whole-cell patch, modelling, and in vitro fictive locomotion studies show the effects of M-currents on locomotion. Interestingly, they show a rather dramatic interaction between IM and INaP where IM works in opposition to INaP to control the duration of bursts and the frequency of pacemaker cells. So overall excellent work uncovering a new mechanism for burst regulation within the locomotor central pattern generator of the spinal cord.**

**Major Comments**

**I was pleased to see the authors show that M-currents contribute to locomotion in vivo, but some issues regarding interpretation remain. The authors acknowledge that manipulation of M-currents using IP injections would affect locomotor centres throughout the brain. What would be useful here, if possible, would be to run a set of experiments using intrathecal injections targeting the L1/2 segments of the spinal cord with the agonists/antagonists. That said I was impressed that the fictive locomotor activity showed responses that were similar to the in vivo situation.**

To meet the reviewer’s concern, additional experiments have been performed. Due to technical constraints related to the size of the animal and that of our catheter, we were unable to perform the experiments in juvenile rodents or mice. To overcome this limitation, in a first set of experiments we tried to perform in juveniles intervertebral injections of drugs but this approach requires at least an anaesthesia of the animal. Unfortunately, the recovery time from this anaesthesia did not allow us to study the acute effect of the drug. Thus, we studied the effects in adult rats implanted with an intrathecal catheter delivering drugs at L1/L2. Because of the difficulty of this set of experiments and to reduce the number of animals, we have focused on the role of Kv7.2/3 subunits in regulating the speed of locomotion by using the potent I_M_-enhancer ICA73 and the I_M_-blocker XE-991. With this new set of experiments, we confirmed data submitted in the first version of the manuscript; i.e that the intrathecal perfusion of drugs at the L1/L2 level replicated the effects observed with an intraperitoneal injection. Specifically, rats walked faster with ICA73 and slower with XE-991. These additional data strengthens the concept that *I*_M_ is an important and physiologically relevant regulator of the spinal locomotor network. These key data are now illustrated in the main Figure 1 and in the supplementary figure S1, added in results (see Page 5 line 19 to page 6 lines 4 in tracked manuscript). Material and methods related to these experiments have been updated. (see Page 23 lines 8-22; Page 37 lines 1-3 in tracked manuscript).

**Minor Comments**

**Results**

**Page 5. Line 20. I think the summary statement that Kv72/3 channels are affecting the clock is something that is best discussed rather that concluded at the early stage of results. There are other possibilities here for fast/slow walking that don't include the clock but could include changes in descending drive.**

We are agree with the reviewer and removed the notion of the clock function from the sentence.

**Page 6. Line 24. Add a citation to the 'electrophysiological signature of IM'.**

We added the reference from Brown and Adam, 1980. Muscarinic suppression of a novel voltage-sensitive K+ current in a vertebrate neurone; Nature. 1980 Feb 14;283(5748):673-6

**Figure 1. Exchange ICA73 and Linopiridine traces in A. That would put the agonist/antagonists pairs in the same row.**

Exchange was done.

**Figure legends. Make it clear species in legends. Fig 3 has both rat and mouse.**

Correction was done in the text.

**Discussion**

**I would have a separate paragraph devoted to the paper unravelling the paradox of how increasing IM which decreases excitability translates to an increase in the rhythmic frequency of walking.**

We think that an indirect interplay between I_M_ and the Na/K pump current regulates the rhythmic frequency of walking. This paradox has been further discussed in the manuscript (see page 18, lines 3-15 tracked manuscript) as following “*This paradox implies that I_M_ indirectly dictates the interburst interval and thus the rhythmic frequency of walking. The Na/K pump current has been linked to the rhythmogenic mechanism of the spinal locomotor central pattern generator networks (Darbon et al., 2003; Zhang and Sillar, 2012; Kueh et al., 2016; Picton et al., 2017). Specifically, the activation of Na/K pumps in response to intracellular Na^+^ accumulation during locomotion, decelerates the frequency of locomotor rhythm by mediating a post-burst hyperpolarization (Picton et al., 2017). Because the decrease of I_M_ increases the duration of Na^+^(I_Nap_)-mediated bursts, we posit that in response to Na^+^ accumulation the subsequent post-burst hyperpolarization mediated by Na/K pump will be higher, and therefore enables neuronal bursting and rhythmic frequency of walking at low frequencies. In sum, the indirect link between I_M_ and Na/K pump through the Na^+^ accumulation during locomotor bursts might finely tunes the frequency of the rhythm by regulating the interburst duration.*”

**Page 15 - line 23. Plead to suggest.**

Correction was done in the text.

**Page 17. Lines 19-20. Reword.**

The sentence has been rephrased as follow: “*The effects of I_M_ on the controllability of the locomotor rhythm in vivo, lead us to conclude that I_M_ provides a potential to adapt speed of movements as circumstances demand*”.

**Page 18. Line 19. Aside from.**

Correction is done in the text.

**Methods.**

**Throughout. It is not very clear why the concentrations of agonists/antagonists used were chosen.**

For *in vitro* experiments, concentrations were chosen based on literature and IC50 or EC50 have been added in the method section in order to justify the selected concentrations. For *in vivo* experiment, the doses have been chosen according to the literature as already cited in the method section “*The dose of drugs refers to previous reports [30-32]*.”

**Reviewer #4: The manuscript by Verneuil et al attempts to define the function of the M-current and KCNQ channels in locomotion CPG, an unexplored and important topic. They authors use an array of tools including a Kcnq2 knockin mouse model of a recurrent pathogenic mutant (KCNQ2T274M) to determine the precise function of KCNQ channels and its relationship to the sodium persistent current in spinal cord interneurons. This work strongly suggests that the M-current regulates the activity of the sodium persistent current, which in turn controls the firing behavior of locomotion interneurons and locomotion CPG in general. Although this work provides a significant advance in our understanding of KCNQ channels in the nervous system, some of the conclusions need to be better flushed out by the authors.**

**Major Comments**

**(1) It is well established that the molecular correlate of the M-current is KCNQ2/3 channels. However, over the years there have been suggestions that KCNQ2 homomeric channels could mediate the M-current. Based on immunostaining of ventromedial interneurons the authors postulate that the M-current in spinal interneurons is likely due to axonal KCNQ2 channels. Unfortunately, the data do not fully support this assertion. First, in Figure 2 there is a substantial signal for both KCNQ2 and KCNQ3 at the soma. The signal is everywhere in the soma. Somatic signal was not quantified. Two, their conclusion is based on non-validated antibodies. Three, 100nM retigabine (Fig S3) increased the holding current and amplitude of the M-current. Retigabine has very high preference for KCNQ3 and KCNQ2/3 channels, over KCNQ2 channels, particularly at 100nM (Schenzer et al 2005 JNS Figure 1). Fourth, the use of KCNQ2 T274M mice suggest that KCNQ2 channels mediate the M-current, but does not exclude the possibility that KCNQ2/3 also mediates it as KCNQ2T274M will also reduce KCNQ2/3 currents.**

**Thus, to better examine whether the KCNQ2 or KCNQ2/3 channels mediate the M-current in spinal interneurons the authors need to test the effect of 5mM TEA on the M-current. 5 mM TEA fully blocks KCNQ2 homomers but only partially KCNQ2/3 channels. Please see Carver et al (Figure 4A JNS 2019) for similar experiments in neurons.**

We agree with the reviewer. It was not our intention to claim that I_M_ from CPG interneurons is exclusively mediated by Kv7.2 channels in homomeric form. As mentioned in the discussion of the original manuscript we did not exclude a contribution of Kv7.3 channels. The principal point established in our experiments is that the presence of the Kv7.2 subunits is important for the expression of functional M-channels in the CPG neurons, as either homomers or as heteromers with Kv7.3 or again as a combination of the two. To avoid any confusion we now refer throughout the manuscript that I_M_ was composed of Kv7.2**-containing** channels.

Our study did not aim at determining the subcellular distribution of functional M-channels and it was not our intention to claim that Kv7.2 channels from the axons exclusively mediate I_M_. This statement in the summary was awkward and has been removed and the immunostaining at the level of the soma is now stated in results.

About the concern on the specificity of antibodies please see the answer to the question 4 from the reviewer 2, and the additional experiments we did perform to validate the Kv7.2 antibody.

As requested by the reviewer, we exploited the differential sensitivity of homomeric and heteromeric Kv7 channels to the K channel blocker TEA (Wang et al., 1998; Hadley et al., 2000, 2003; Schroeder et al., 2000; Shapiro et al., 2000). Homomeric Kv7.2 and Kv7.3 channels are blocked with IC50 values of 0.17, 200 mM, respectively. Heteromeric channels show intermediate sensitivities to TEA block. Specifically, Kv7.2/7.3 channels have IC50 values near 3 mM. To meet this reviewer’s concern, additional experiments have been performed with 1 mM TEA. We found that a bath application of 1 mM TEA almost abolished the M-current in L1/L2 ventromedial interneurons. The data could be interpreted by assuming that some of the current could be carried by homomeric Kv7.2 channels in neonatal neurons. However, it is worth considering that a late developmental expression of Kv7.3 leading to a progressive switch towards heteromeric Kv7.2/7.3 in adulthood might occur (Tinel et al., 1998; Hadley et al., 2003). This new data is now added in results (see page 8, lines 1-9 in tracked manuscript) illustrated (see Fig 4) and discussed (see page 15 line 22 to page 16 line 5 in tracked manuscript).

**2. The M-current values only reflect a fraction of the total KCNQ mediated currents in neurons. The authors, rather than reporting only M-current values (deactivating component), they should also report the total XE991 sensitive current that includes the faster KCNQ2/3 current component in Figure 3. Additionally, the authors should also show the re-activating component of the current (the return step).**

Unfortunately, the figure 3 charactering I_M_ is already dense and adding the re-activating component to all recordings made it unreadable. To meet the reviewer request we only illustrate this component in the initial recordings illustrated in Fig 3A. Likewise the total XE991 sensitive current has been added for recordings in Hb9 cells and illustrated in S5A Fig.

**3. The G-V shifts using the ICA73 are difficult to understand (Figure 3). They authors show that application of ICA73 increases the holding current, likely, due to opening of KCNQ2 mediated M-current. This is consistent with the established ICA73 mechanism of action, which is to cause a left shift to the KCNQ2 channels G-V (Wang et al 2018 JGP). However, the authors do not see a significant shift in the G-V. Has the ICA application alter kinetics of the M-current as it usually does (Wang et al 2018 JGP)? Please clarify why ICA73 does not shift the G-V to hyperpolarized Vm.**

There is likely a misunderstanding. As stipulate in the original manuscript the *I*_M_-enhancer ICA73 significantly hyperpolarized the V_T_ and V_1/2_ activation of *I*_M_ suggesting that the ICA73 shifted the G-V curve to hyperpolarized Vm.

**4. The authors did not find any effect of XE991 in the spiking activity of ventromedial interneurons. The attributed this to the poor blocking effect of XE991 at resting membrane potentials. This poor blocking issue can be bypassed by prolonging the application time of XE991 as shown by Hu and Bean (Neuron 2018). Therefore, the authors should repeat the experiments in Figure 4B using a 30 minute application of XE991.**

This is the time window of application we used in our experiments. This is now specified in the results as following ”*The* I*_m_-blocker per se neither depolarised the resting membrane potential nor increased the neuronal excitability or the firing rate even 30 min after the bath application of the drug”*

**5. Because of the lack of effect of XE991 in ventromedial interneurons the authors chose to use modeling. It would have been better to use the Kcnq2 T274M mice. Consequently, the authors need to report whether the firing properties of ventromedial interneurons are increased in Kcnq2T274M mice. This will strengthen their conclusions over modeling, which is based on several assumptions.**

We agree with the reviewer. To meet the reviewer concern we performed additional intracellular recordings on Kv7.2^Thr274Met/+^ mutant mice. As predicted by the model, we showed that ventromedial interneurons from mutant mice, displayed a higher spiking frequency. These data are now illustrated in the figure 4 (see Fig. 4C-E) and added in results (page 9, lines 9-12 in tracked manuscript).

**6. The key experiment in this manuscript is to show whether the sodium persistent current has increased in Kcnq2T274M mice and whether Kcnq2T274M mice have gait or locomotion coordination issues as shown in Figure 1 using pharmacology.**

As requested by the reviewer, we characterized I_NaP_ in ventromedial interneurons from neonatal Kv7.2^Thr274Met/+^ mutant mice. We found a more pronounced I_NaP_ in mutant mice compared to wildtype animals. These data are now illustrated in the figure 6C and D, and added in results (see Page 11, lines 1-2 in tracked manuscript). These results are consistent with a slower fictive locomotor activity recorded in mutant animals (Fig. 8). Indeed, we previously demonstrated that a specific upregulation of *I*NaP by veratridine slowed down the locomotor rhythm (Tazerart et al., 2008). However, locomotor performances measured in juvenile Kv7.2^Thr274Met/+^ mutant mice when they acquired a full-weight-bearing stepping, appeared similar to that observed in wildtype animals. This new data has been added at the end of results and illustrated (see S10 Fig). This discrepancy compared to in vitro experiments has been discussed (see Page 16, lines 1-5 in tracked manuscript) and might be due to the fact that data were captured at two different time-window investigations, in neonates for fictive locomotion and juveniles for real locomotion, suggesting that a compensatory mechanism might take place in the knock-in mice with age. In line with this assumption Kv7.2^Thr274Met/+^ mutant mice displayed early epileptic seizures that were generally not observed when animals grew (Milh et al., 2020).

**Minor comments.**

**1. The title does not convey clearly the conclusions of the paper. It is more appropriate for a commentary.**

We believe that the title is in line with the main conclusion of the paper.

**2. The abstract needs to be revised and remove the statement about axonal KCNQ2 channels. The work does not distinguish between the roles of axonal to somatic KCNQ2 channels.**

Please see the answer to the question 1.

**3. For statistics the authors need to report number of cells, slices, and number of animals used for each experiment.**

All numbers of cells, slices, and animals are stipulated for each experiment in figure legends.

Reference List

Adams PR, Brown DA (1982) Synaptic inhibition of the M-current: slow excitatory post-synaptic potential mechanism in bullfrog sympathetic neurones. J Physiol 332:263-272.

Anglister L, Etlin A, Finkel E, Durrant AR, Lev-Tov A (2008) Cholinesterases in development and disease. Chem Biol Interact 175:92-100.

Barber RP, Phelps PE, Houser CR, Crawford GD, Salvaterra PM, Vaughn JE (1984) The morphology and distribution of neurons containing choline acetyltransferase in the adult rat spinal cord: an immunocytochemical study. J Comp Neurol 229:329-346.

Brown DA, Adams PR (1980) Muscarinic suppression of a novel voltage-sensitive K+ current in a vertebrate neurone. Nature 283:673-676.

Carlin KP, Dai Y, Jordan LM (2006) Cholinergic and Serotonergic Excitation of Ascending Commissural Neurons in the Thoraco-Lumbar Spinal Cord of the Neonatal Mouse. Journal of Neurophysiology 95:1278-1284.

Darbon P, Tscherter A, Yvon C, Streit J (2003) Role of the electrogenic Na/K pump in disinhibition-induced bursting in cultured spinal networks. J Neurophysiol 90:3119-3129.

Delmas P, Brown DA (2005) Pathways modulating neural KCNQ/M (Kv7) potassium channels. Nat Rev Neurosci 6:850-862.

Etlin A, Finkel E, Cherniak M, Lev-Tov A, Anglister L (2014) The motor output of hindlimb innervating segments of the spinal cord is modulated by cholinergic activation of rostrally projecting sacral relay neurons. J Mol Neurosci 53:517-524.

Greene DL, Hoshi N (2017) Modulation of Kv7 channels and excitability in the brain. Cell Mol Life Sci 74:495-508.

Hadley JK, Passmore GM, Tatulian L, Al-Qatari M, Ye F, Wickenden AD, Brown DA (2003) Stoichiometry of expressed KCNQ2/KCNQ3 potassium channels and subunit composition of native ganglionic M channels deduced from block by tetraethylammonium. J Neurosci 23:5012-5019.

Huang A, Noga BR, Carr PA, Fedirchuk B, Jordan LM (2000) Spinal cholinergic neurons activated during locomotion: localization and electrophysiological characterization. J Neurophysiol 83:3537-3547.

Jordan LM, McVagh JR, Noga BR, Cabaj AM, Majczynski H, Slawinska U, Provencher J, Leblond H, Rossignol S (2014) Cholinergic mechanisms in spinal locomotion-potential target for rehabilitation approaches. Front Neural Circuits 8:132.

Kiehn O, Johnson BR, Raastad M (1996) Plateau properties in mammalian spinal interneurons during transmitter-induced locomotor activity. Neuroscience 75:263-273.

Krey RA, Goodreau AM, Arnold TB, Del Negro CA (2010) Outward Currents Contributing to Inspiratory Burst Termination in preBotzinger Complex Neurons of Neonatal Mice Studied in Vitro. Front Neural Circuits 4:124.

Kueh D, Barnett WH, Cymbalyuk GS, Calabrese RL (2016) Na(+)/K(+) pump interacts with the h-current to control bursting activity in central pattern generator neurons of leeches. Elife 5.

Legendre P, McKenzie JS, Dupouy B, Vincent JD (1985) Evidence for bursting pacemaker neurones in cultured spinal cord cells. Neuroscience 16:753-767.

Marrion NV (1997) Control of M-current. Annu Rev Physiol 59:483-504.

Miles GB, Hartley R, Todd AJ, Brownstone RM (2007) Spinal cholinergic interneurons regulate the excitability of motoneurons during locomotion. Proc Natl Acad Sci U S A 104:2448-2453.

Picton LD, Nascimento F, Broadhead MJ, Sillar KT, Miles GB (2017) Sodium Pumps Mediate Activity-Dependent Changes in Mammalian Motor Networks. J Neurosci 37:906-921.

Purvis LK, Smith JC, Koizumi H, Butera RJ (2007) Intrinsic bursters increase the robustness of rhythm generation in an excitatory network. J Neurophysiol 97:1515-1526.

Selyanko AA, Hadley JK, Wood IC, Abogadie FC, Jentsch TJ, Brown DA (2000) Inhibition of KCNQ1-4 potassium channels expressed in mammalian cells via M1 muscarinic acetylcholine receptors. J Physiol 522 Pt 3:349-355.

Tillakaratne NJ, Duru P, Fujino H, Zhong H, Xiao MS, Edgerton VR, Roy RR (2014) Identification of interneurons activated at different inclines during treadmill locomotion in adult rats. J Neurosci Res 92:1714-1722.

Tinel N, Lauritzen I, Chouabe C, Lazdunski M, Borsotto M (1998) The KCNQ2 potassium channel: splice variants, functional and developmental expression. Brain localization and comparison with KCNQ3. FEBS Lett 438:171-176.

Zagoraiou L, Akay T, Martin JF, Brownstone RM, Jessell TM, Miles GB (2009) A cluster of cholinergic premotor interneurons modulates mouse locomotor activity. Neuron 64:645-662.

Zhang HY, Sillar KT (2012) Short-term memory of motor network performance via activity-dependent potentiation of Na+/K+ pump function. Curr Biol 22:526-531.
