# Supplementary material for: The M-current works in tandem with the persistent sodium current to set the speed of locomotion
Source: PLoS Biol. 2020 Nov 13;18(11):e3000738. doi: 10.1371/journal.pbio.3000738 (PMC7688130; doi:10.1371/journal.pbio.3000738)
Supplement: Attachment — Submitted filename: Response to reviewers.doc [file pbio.3000738.s013.doc]

September 24, 2020

Manuscript Number: PBIOLOGY-D-20-00922R2

Dear Editor,

After minor revision, we submit the new version of our manuscript PBIOLOGY-D-20-00922R2 for publication as a Research Article in Plos Biology. We would like to thank the reviewers for their helpful suggestions and the editors for their interest regarding the manuscript. We believe that we have addressed all requirements from the editor as well as the production requirements to conform the paper to guidelines. Text that was revised is highlighted in red.

Thank you again for accepting our manuscript for publication in PLOS Biology.

Sincerely,

 Frédéric Brocard.
